# Predicting the mortality of patients with Covid‐19 A machine learning approach: Correspondence

**DOI:** 10.1002/hsr2.1381

**Published:** 2023-09-12

**Authors:** Seyed Mohammad Ayyoubzadeh

**Affiliations:** ^1^ Medical Informatics, Department of Health Information Management, School of Allied Medical Sciences Tehran University of Medical Sciences Tehran Iran

Dear Editor,

I would like to discuss the article recently published entitled “Predicting the mortality of patients with Covid‐19: A machine learning approach”.^1^ This work is done by training classifiers (Random Forest, Regression Logistic, Gradient Boosted Trees, and Support Vector Machine) on a relatively big data set from five hospitals. The First and major concern raised is: it seems in Figure 4B there is a clear cut based on the D‐Dimer test that could be used to determine the class (Discharge and Death). If Figure 4B is representative of the data it seems that the performance of a simple rule (if D‐Dimer > 500 then class = Death else class=Discharge) should outperform the classifiers introduce in the manuscript. Second, in the preprocessing section, the authors stated they have used the KNN technique for replacing missing values however the K is mentioned to be 30 and (simultaneously) 50. It is not clear how the authors used two K numbers (30 and 50) for this purpose although the number of the K is better to be an odd number that the majority vote of neighbors was clear (if the distance does not affect the weight). Third, In the limitation part, the authors stated that one of the limitations of the study is that it is conducted on one database while in the methods section in the samples definition sub‐section, it is noted that the data set contains the data of five hospitals and it seems this limitation is not valid.

## AUTHOR CONTRIBUTIONS

**Seyed Mohammad Ayyoubzadeh**: Conceptualization; writing—original draft; writing—review and editing.

## CONFLICT OF INTEREST STATEMENT

The author declares no conflict of interest.

## Data Availability

Data available on request from the authors.
